# Dual-channel near-field control by polarizations using isotropic and inhomogeneous metasurface

**DOI:** 10.1038/srep15853

**Published:** 2015-11-03

**Authors:** Xiang Wan, Ben Geng Cai, Yun Bo Li, Tie Jun Cui

**Affiliations:** 1 State Key Laboratory of Millimetre Waves, School of Information Science and Engineering, Southeast University, Nanjing 210096, China

## Abstract

We propose a method for dual-channel near-field manipulations by designing isotropic but inhomogeneous metasurfaces. As example, we present a dual-channel near-field focusing metasurface device. When the device is driven by surface waves from different channels on the metasurface, the near fields will be focused at the same spatial point with different polarizations. Conversely, if a linearly polarized source is radiated at the spatial focal point, different channels will be evoked on the metasurface controlled by polarization. We fabricated and measured the metasurface device in the microwave frequency. Well agreements between the simulation and measurement results are observed. The proposed method exhibits great flexibility in controlling the surface waves and spatial waves simultaneously. It is expected that the proposed method and dual-channel device will facilitate the manipulation of near electromagnetic or optical waves in different frequency regimes.

The exploration of electromagnetic near fields dates back to the proposal^1^ and the subsequent experiments^2^,^3^,^4^ of detecting near fields to obtain resolutions beyond the diffraction limit. In recent years, great efforts have been dedicated to the near-field focusing in microwave^5^,^6^,^7^,^8^, terahertz^9^,^10^,^11^,^12^ and optical frequencies^13^,^14^,^15^, which inspired many applications such as the sub-wavelength imaging^16^,^17^,^18^,^19^,^20^ and near-field probing^13^,^21^,^22^,^23^. Besides focusing the near fields, other methods of manipulating near fields have also been established, including the transformation optics^24^,^25^,^26^ and generalized Snell’s law^27^,^28^,^29^, which dramatically promote the utilizations of the electromagnetic near fields.

Generally, these manipulating processes start with impinging waves and end with scattering waves. The radiation source, the manipulation device, and the detector constitute the whole processing system, which is inevitably space-consuming. On the contrary, plasmonics deals with the tightly confined electromagnetic surface waves on planes or curved surfaces^30^,^31^,^32^,^33^,^34^. By means of the surface waves, more compact systems for manipulating near fields have been obtained. For instance, one can design planar microscopes or detectors since the sources can be integrated with the manipulating devices. In other words, the inputs for these devices are surface waves and the outputs are spatial waves, and vice versa. In this case, the manipulating devices behave as couplers for the two kinds of electromagnetic waves. Several methods have been developed to directionally excite the surface waves by near fields^35^ or focus the near fields by leaky-wave sources^36^,^37^. The manipulations of the far fields are also implemented by sinusolidlly modulating^38^,^39^ or holographically modulating surface impedances of metasurfaces^40^,^41^,^42^.

In this paper, a method based on holographic modulations is proposed to manipulate near fields in dual channels by designing isotropic and inhomogeneous metasurfaces. In particular, we present a dual-channel metasurface to focusing the fields in the Fresnel zone. The input of the device can be either a directional or cylindrical surface wave, while the output is the focused leaky wave with specific polarization depending on the input source. Conversely, when the input is a linearly polarized source at the spatial focal point, the output will be the directional or cylindrical surface wave along different channels on the metasurface, depending on the polarization of the source. Actually, different polarizations at the focal point ensure the dual-channel characteristic of the presented device. Verification experiments are performed in the microwave frequency, and well agreements are observed between simulations and experiments.

## Theoretical Method

**Figure 1:**
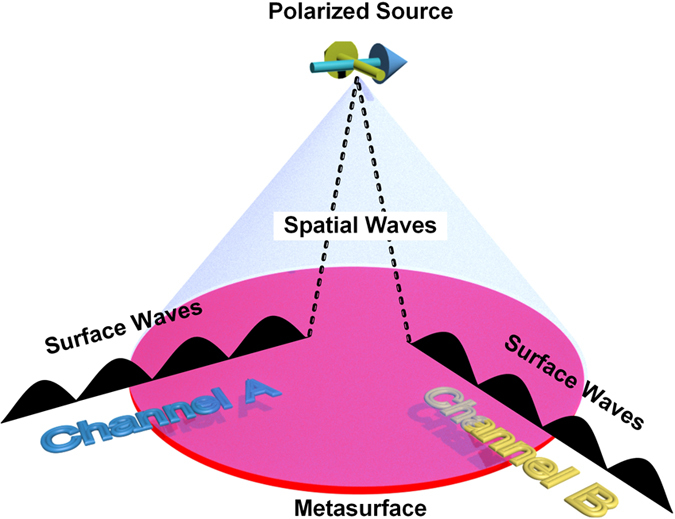
A conceptual diagram of the dual-channel metasurface device.

In order to depict the concept more clearly, a conceptual diagram of the dual-channel metasurface device is drawn in Fig. 1. Different polarizations of the radiation sources at the focal point will create orthogonal channels on the metasurface. When the metasurface device is driven by surface waves along different channels, the near fields will converge at the same spatial point outside the metasurface with different polarizations. Conversely, if a linearly-polarized source is placed at the focal point, directional or cylindrical surface waves are excited along different channels, depending on the source polarization. From the perspective of realization, it is naturally to construct anisotropic metamaterials for dealing with polarization-sensitive problems. However, we present here an isotropic but inhomogeneous metasurface to design the polarization-dependent dual-channel device.

**Figure 2:**
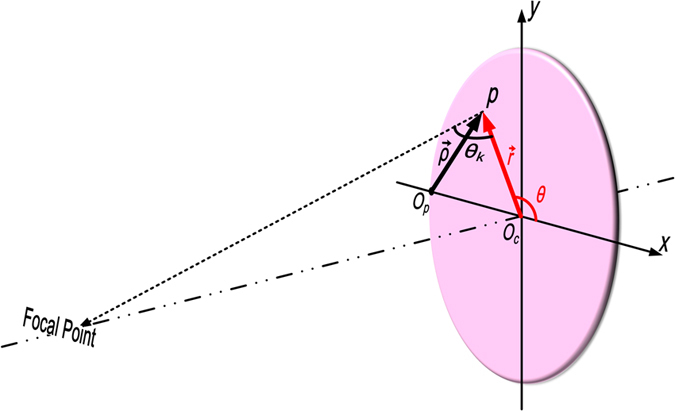
The schematic to design the isotropic and inhomogeneous metasurface.

For specification, we design a dual-channel near-field focusing device using an isotropic and inhomogeneous metasurface. Considering that a point source is placed at point *O*_*p*_ (see Fig. 2) to excite TM-mode surface waves on the uniform plate, then the equivalent surface current and tangent electric field can be interpreted as


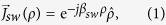



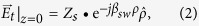


where *β*_*sw*_ is the surface wavenumber; *Z*_*s*_ is the surface impedance and ρ is the distance from the point *O*_*p*_to the point *P.* As the present design focuses the phase information of the source, hence these expressions have ignoring the amplitude attenuation term (1/ρ^2^) of the surface current. Becasue *β*_*sw*_ is greater than the wavenumber (*k*_*0*_) in free space, the waves in Eq. (2) are confined on the plate. To produce leaky waves, the value of wavenumber is altered by modulating the surface impedance. The formula of the modulation is expressed as


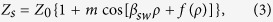


in which *Z*_0_ is the averaged surface impedance, *m* is the modulation index, and *f* (*ρ*) is the state function. The carrier wave in Eq. (3) is the cylindrical surface wave with wavenumber *β*_*sw*_. Replacing the cosine function in Eq. (3) by exponential functions, the equivalent surface current and tangent electric field change to


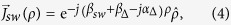



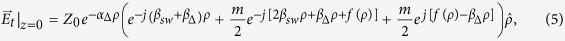


where *β*_∆_*-jα*_∆_ is the perturbation term of the wavenumber. Given that 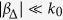, if 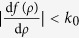, the first two terms in the parentheses of Eq. (5) are surface waves, and the leaky waves are produced only by the third term.

Since the leaky waves are supposed to be focused in the space outside the metasurface, the state function *f* (*ρ*) is determined by the phase matching condition, that is, the transverse phase gradient of the state function satisfies the following condition


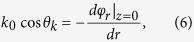


where *φ*_*r*_|_z=0_ = *f*(*ρ*) − *β*_∆_*ρ*; 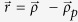 and 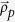 points to *O*_*c*_ from *O*_*p*_; *θ*_*k*_ is the elevation angle for each point on the plate, as shown in Fig. 2. If the vertical distance between the focal point and metasurface is *h*, we have


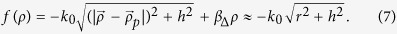


In the Cartesian coordinate with the origin being point *O*_*c*_, the third term in Eq. (5) is expressed as


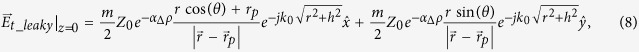


where *θ* is the azimuth angle for each point on the plate and 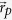 points to *O*_*p*_ from *O*_*c*_ . Based on vector diffraction theory^43^, the horizontal components of electric field along the focal axis are evaluated by


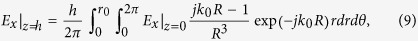



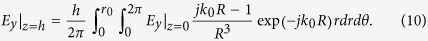


where 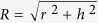. By observing the integrations, we find that *E*_*x*_ ≠ 0 and *E*_*y*_ = 0. More importantly, the integrations are independent on the carrier waves. As a result, the near-field focusing is enabled by different sources. For instance, if a directional source drives the metasurface along the *y* direction, the modulation function of the surface impedance and the horizontal components of electric field along the focal axis become


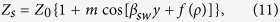



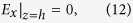



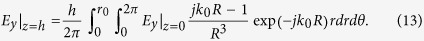


We then have *E*_*x*_ = 0 and *E*_*y*_ ≠ 0 at the focal point. Since the modulation of the surface impedance is linear, Eqs. (3) and (11) can be compounded to form a hybrid modulation function


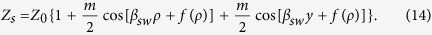


**Figure 3:**
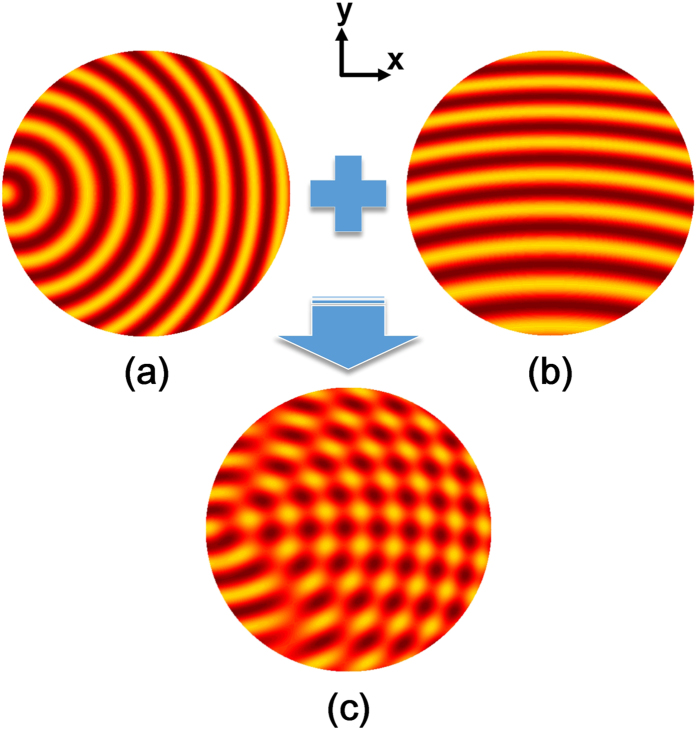
Process of superimposing the impedance modulation at 15 GHz. The original point locates at the left end of the circular area. (**a**) The impedance pattern for cylindrical carrier waves with Z_*s_c*_ = *j*177.1(1 + 0.2cos(1.1*k*_0_*r*_*s*_ + *k*_0_*r*_*f*_)), where 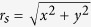; 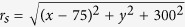. (**b**) The impedance pattern for directional carrier waves with Z_*s_d*_ = *j*177.1(1 + 0.2cos(1.1*k*_0_*y* + *k*_0_*r*_*f*_)). (**c**) The hybrid impedance pattern for the dual carrier waves with Z_*s_h*_ = (Z_*s_c*_ + Z_*s_d*_)/2.

Figure 3 demonstrates the process of the superposition, in which (a) and (b) are the impedance patterns when the carrier waves are cylindrical surface waves and directional surface waves, respectively, and (c) is the hybrid impedance pattern. To excite the inhomogeneous metasurface is essentially to demodulate the state function *f* (*ρ*) from the impedance modulation function. Referring to Figs 1 and 3, we notice that the leaky waves are focused with *E*_*y*_ = 0 in channel A, when a point source is used to feed the metasurface from the left side in the *x* direction; while the leaky waves are focused with *E*_*x*_ = 0 in channel B, when a directional source is used to feed the metasurface from the bottom side in the *y* direction. For the convenience of experiments in the presented design, the state function is chosen to focus the leaky waves for both carrier waves. Actually, the state functions for different carrier waves in Eq. (14) are independent from each other, which means that we can focus the leaky waves in channel A, meanwhile steer a directional beam in channel B.

## Design and Experiments

**Figure 4:**
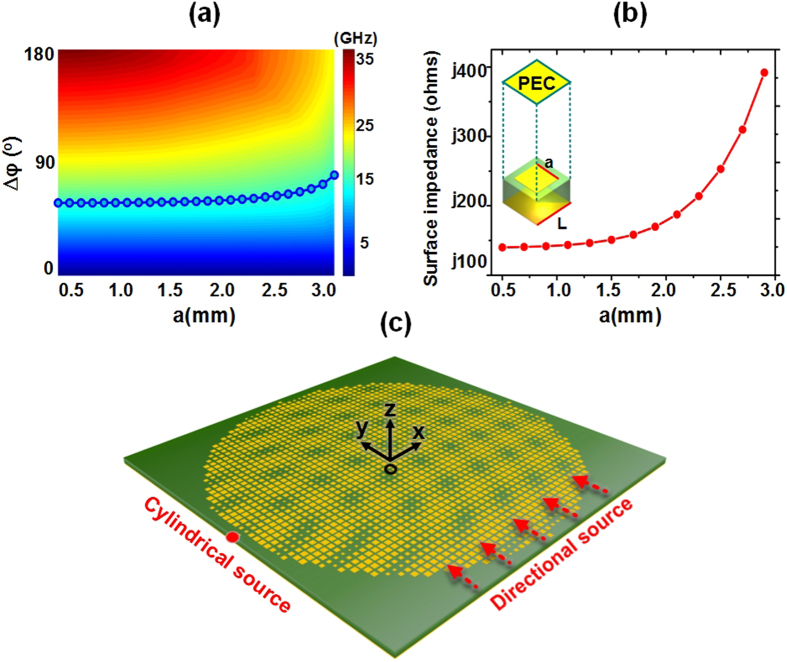
The process of designing the metasurface. (**a**) Dispersion map of the unit cell shown in the inset of Fig. 4(b). (**b**) The relationship of the surface impedance and the patch width *a*. The period of the unit is *L* = 3 mm. In the Eigen-mode simulations of the unit, periodic boundaries are used on four sides, and an electric wall is placed on the top of the unit. (**c**) The geometric structure of the metasurface. The diameter of the modulated area is 150 mm, and the size of the substrate is 160 mm*160 mm. In the full-wave simulations, the cylindrical source and directional source are used individually.

The proposed metasurface device is designed on a grounded print circuit board with square metal patches on the top side, as illustrated in Fig. 4(c) and the inset of Fig. 4(b). By virtue of the commercial software, CST Microwave Studio, we numerically obtain the Eigen frequencies of differently sized metal patches. During the simulations, periodic boundaries are used in the four sides, and perfect electrical conductor (PEC) is placed on the top of the unit cell, as shown in the inset of Fig. 4(b). The periodic *L* of the unit cell is 3 mm. Figure 4(a) illustrates the dispersion map, in which the dotted line presents the relation between the patch width (*a*) and the phase variation (*∆φ*). For the TM-mode surface waves, the surface impedances is derived by the following equation


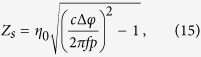


which leads to the relation curve demonstrated in Fig. 4(b). Once we establish the mapping relationship between the surface impedance *Z*_*s*_ and the patch width *a*, the impedance pattern given in Eq. (14) (see Fig. 3(c)) can be implemented by varying the patch width. In the numerical calculations, we set the average impedance as *Z*_0_ = *j*177.1 Ω, the working frequency as *f* = 15 GHz, the modulation index as *m* = 0.2, and the height of the focal point as *h* = 300 mm. By such designs, the dual-channel metasurface device for near-field manipulation is realized. Figure 4(c) displays the structure diagram of the isotropic and inhomogeneous metasurface, which is used to perform the full-wave simulations by CST.

**Figure 5:**
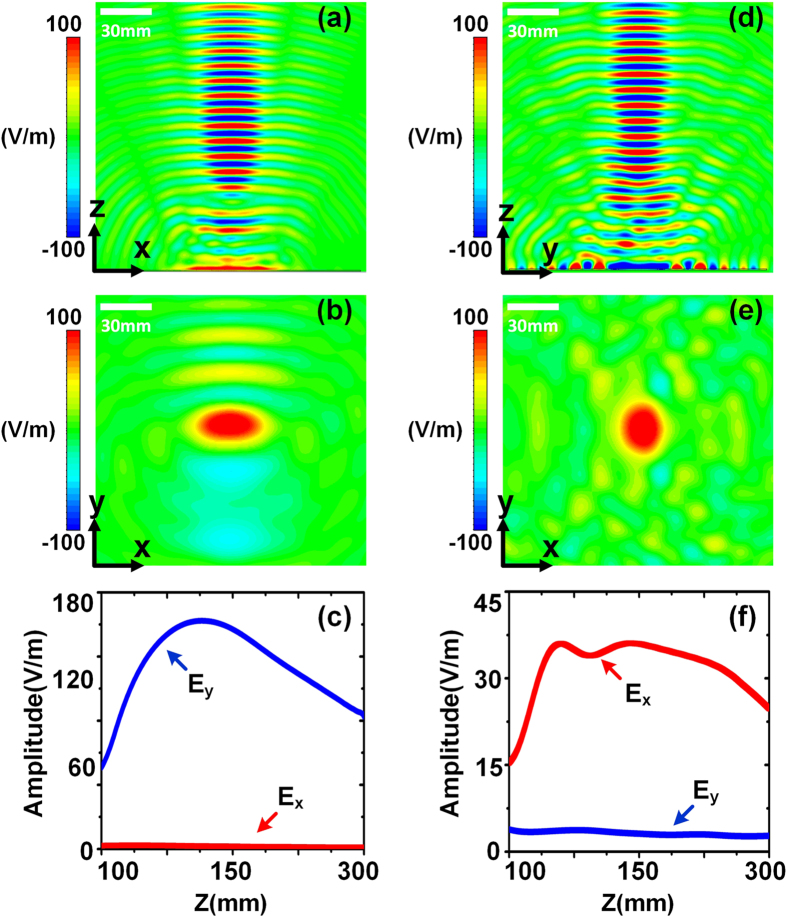
Full-wave simulation results under different exciting sources. (**a**–**c**) Simulation results under the excitation of directional source. (**a**) The *x* components of electric fields on the vertical section. (**b**) The *y* components of electric fields on the horizontal section. (**c**) The comparison between the amplitudes of *E*_*x*_ and *E*_*y*_ along the focal axis. (**d**–**f**) Simulation results under the excitation of cylindrical source. (**d**) The *y* components of electric fields on the vertical section. (**e**) The *x* components of electric fields on the horizontal section. (**f**) The comparison between the amplitudes of *E*_*x*_ and *E*_*y*_ along the focal axis.

Since the modulation function of the surface impedance contains two kinds of carrier waves that correspond to particular sources, the metasurface is differently motivated in the full-wave simulations. Firstly, a dipole source is place on point A (see Fig. 4(c)) to excite the cylindrical surface waves on the metasurface. Figure 5(a,b) present numerical results of *x* components of electric fields on the vertical and horizontal sections, respectively. We clearly observe that the leaky waves are focused in the air. The magnitudes of electric fields along the focusing axis are extracted from the simulated results, as illustrated in Fig. 5(c). We note that the *x* components of electric fields play a dominant role in the focusing axis.

Secondly, directional surface waves are used along the *y* direction to excite the metasurface (see Fig. 4(c)). As expected, the leaky waves are likewise focused in the air. Figure 5(d,e) demonstrate the numerical results similar to those when the point source is used. However, Fig. 5(f) shows that the *y* components of electric fields are much large than the *x* components, which means that the leaky fields on the focal point possess different polarizations when different driving sources are adopted. The above simulations clearly verify the theoretical analyses on the polarization characteristics of the fields at the focal point. The displacement between the location of the maximum intensity and designed focal point (*h* = 300 mm) is resulted from the focal shift phenomenon^44^,^45^. The amplitude difference between Fig. 5(c,f) is resulted from the different excitation efficiencies. For cylindrical waves, a monopole antenna is used to excite the metasurface; for directional waves, an open-end rectangular waveguide is used to excite the metasurface. The excitation efficiency in the latter case is higher than that in the former case, hence the amplitude of *E*_*y*_ is large than that of *E*_*x*_.

**Figure 6:**
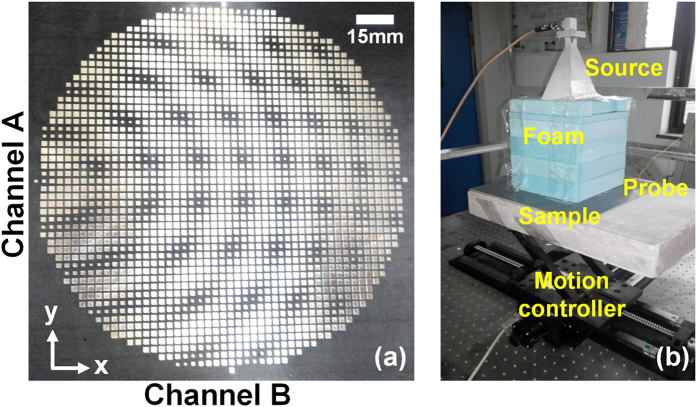
The fabricated sample of the dual-channel metasurface device and the testing environment. (**a**) The fabricated sample of the metasurface. (**b**) The testing environment. A linearly polarized horn antenna is used as the impinging source. With the motion controller, an electric-dipole probe on the top of the sample records the pattern of electric fields.

**Figure 7:**
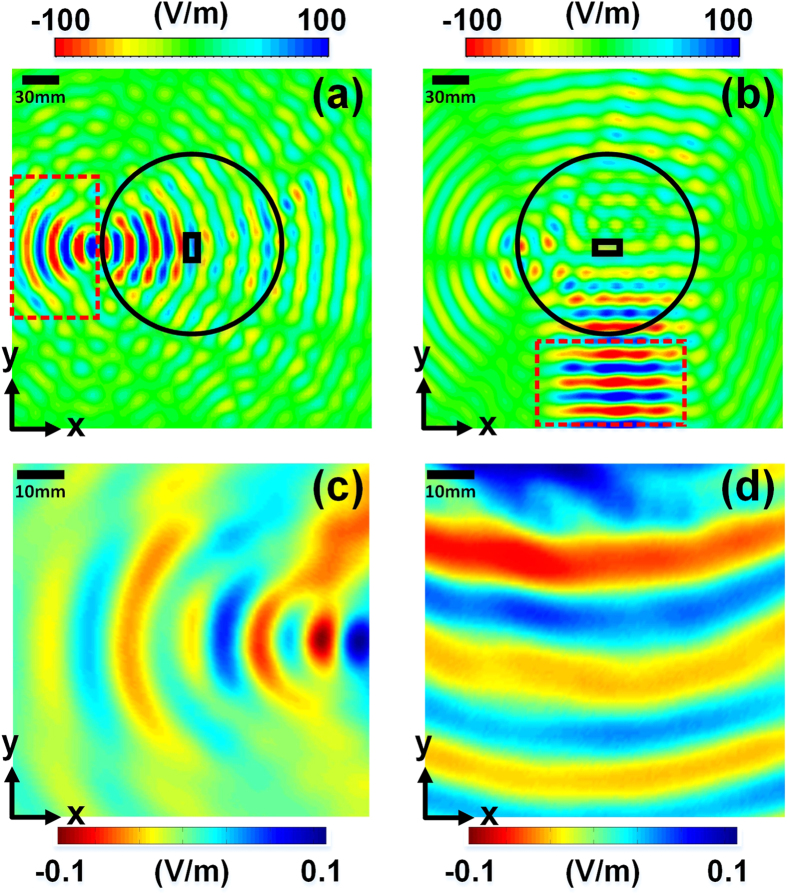
Simulated and measured results of near electric fields on the metasurface excited by a linearly polarized horn antenna located at the focal point, in which the black solid circular and rectangular lines represent the metasurface and feeding waveguide for the horn antenna, while the red dashed rectangular indicate the measurement domain. (**a**,**b**) The simulated patterns of electric fields on the top of the metasurface when the horn antenna is polarized in the *x* and *y* directions. (**c**,**d**) The measured patterns of electric fields on the top of the metasurface when the horn antenna is polarized in the *x* and *y* directions.

The fabricated sample of the dual-channel metasurface device is shown in Fig. 6(a). To verify the near-field manipulation properties of the device, we design an experiment illustrated in Fig. 6(b). A linearly polarized horn antenna is used to radiate spatial electromagnetic waves on the metasurface device. The space under the horn is filled by foams which approximate to the air. According to the principle of reciprocity, different channels will be evoked when the polarization of the horn changes. An electric-dipole probe is used to record the magnitudes of the near electric fields on the metasurface. The horn and the probe are connected to a vector network analyzer (VNA, Agilent N5230C). By dealing with the amplitudes and phases of the scattering parameters (S_21_), the pattern of electric fields is recovered. Figure 7(a,b) display the CST full-wave simulation results, from which we clearly observe the focused surface waves in the horizontal channel (channel A) and directional surface waves in the vertical channel (channel B) when the polarization direction of the horn antenna changes. Figure 7(c,d) provide the measured near-field results in the measurement domain (see Fig. 7(a,b)) under the corresponding polarizations, showing the focused and directional surface waves in channels A and B, respectively. Within the measurement domain, the simulation and experiment results have good agreements, demonstrating the dual-channel properties.

## Discussions

For the presented focusing device, it is very difficult to estimate the exact focusing efficiency in both the simulations and measurement, because energy will be reflected and divulged when we excite the metasurface with different sources, hence the exact focusing energy is hard to be obtained. However, we can use the modulation index to estimate the focusing efficiency. In Eq. (5), the focusing term is (*m*/*2*)exp{−*j*[2*β*_*sw*_*ρ* + *β*_*∆*_*ρ* + *f*(*ρ*)]}, then the focusing efficiency can be estimated by *η* = *m*^*2*^/(4 + 2*m*^*2*^). In the manuscript, the modulation index *m* = 0.2, hence the focusing efficiency is estimated as *η* = 1%.

**Figure 8:**
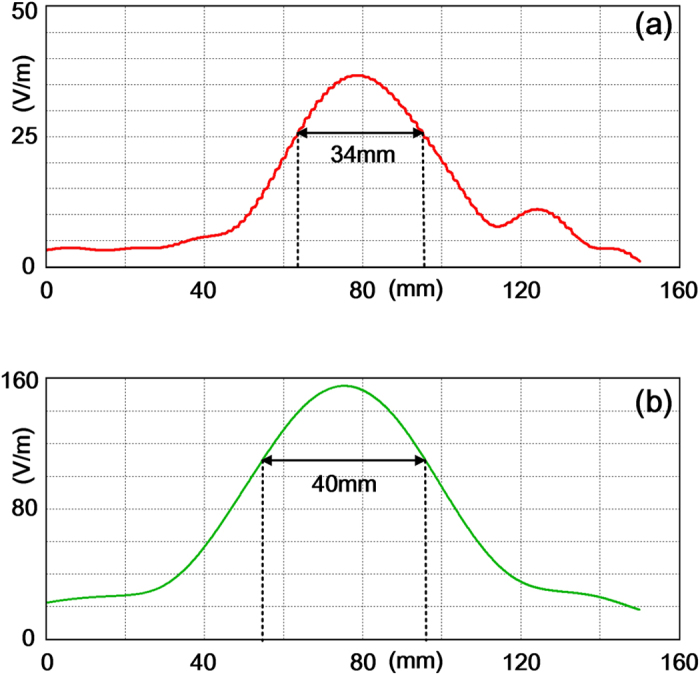
Extracted electric field along a transverse line which passes through the focal point. (**a**) Extracted *E*_*x*_. The arrowed line shows the half power focusing width of *E*_*x*_. (**b**) Extracted *E*_*y*_. The arrowed line shows the half power focusing width *E*_*y*_.

Another key factor of the focusing device is the half power focusing width. The focused electric fields are extracted along transversal lines when different sources are used to excite the metasurface at 15 GHz. Figure 8(a) shows that the half power focusing width for *E*_*x*_ is 34 mm, while Fig. 8(b) shows the width for *E*_*y*_ is 40 mm. Both values are bigger than the working wavelength (20 mm), indicating that the presented device is failed to realize a subwavelength focus.

Generally, the holographical modulation methods can be divided into two types. The first type is direct modulation, in which the reference wave and the object wave are directly interfered to produce the holographic surface impedance; the second type is indirect modulation, in which the modulated impedance is inversely calculated from the object wave. The proposed method is also a kind of direct modulation, which is used to realize near-field focusing. The biggest difference is that the proposed method can deal with differently shaped waves in the near field by choosing adequate modulation term (*f*(*ρ*)). Besides, we have shown that the presented method is robust in simultaneously dealing with near fields.

## Conclusion

In summary, we have proposed a general method to manipulate near fields in dual channels using isotropic but inhomogeneous metasurface. In particular, we presented a dual-channel metasurface device for near-field focusing controlled by polarization, which has been verified numerically and experimentally in the microwave frequency. Numerical simulations and measured results have good agreements to the theoretical designs. The proposed method exhibits great flexibility in dealing with surface waves and spatial waves simultaneously, and the fabrication of the device is very convenient since the unit of the metasurface is isotropic. The presented method will facilitate the applications of metasurfaces, such as in surface waves processing, coplanar-source imaging and detecting in different frequency regimes.

## Additional Information

**How to cite this article**: Wan, X. *et al.* Dual-channel near-field control by polarizations using isotropic and inhomogeneous metasurface. *Sci. Rep.* **5**, 15853; doi: 10.1038/srep15853 (2015).
